# Restoring hemidesmosomes to prevent cancer cell invasiveness

**DOI:** 10.18632/oncotarget.1196

**Published:** 2013-07-19

**Authors:** S Pyronnet, J Guillermet-Guibert, C Bousquet

**Affiliations:** INSERM UMR 1037, Equipe labellisée Ligue Nationale Contre le Cancer (LNCC), Laboratoire d'excellence Toulouse Cancer (labex TOUCAN), Centre de Recherche en Cancérologie de Toulouse (CRCT), Toulouse, France; Université Toulouse III Paul Sabatier, Toulouse, France.; INSERM UMR 1037, Equipe labellisée Ligue Nationale Contre le Cancer (LNCC), Laboratoire d'excellence Toulouse Cancer (labex TOUCAN), Centre de Recherche en Cancérologie de Toulouse (CRCT), Toulouse, France; Université Toulouse III Paul Sabatier, Toulouse, France.; INSERM UMR 1037, Equipe labellisée Ligue Nationale Contre le Cancer (LNCC), Laboratoire d'excellence Toulouse Cancer (labex TOUCAN), Centre de Recherche en Cancérologie de Toulouse (CRCT), Toulouse, France; Université Toulouse III Paul Sabatier, Toulouse, France.

**Figure 1:**
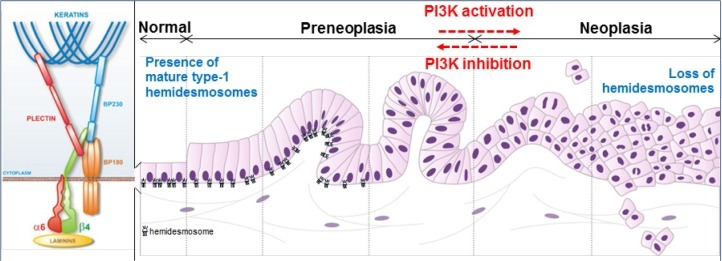
PI3K-dependent loss of hemidesmosomes during pancreatic tumorigenesis Pancreatic tumorigenesis progresses from preneoplasic to neoplasic lesions. Mature type-1 hemidesmosomes anchor epithelial pancreatic duct cells to the underlying basement membrane: transmembrane integrin α6β4 and BP180 (bullous pemphigoid) bind to laminins in the basement membrane, and intracellular hemidesmosome stabilization occurs via their association with keratins through the two plakins, plectin and BP230. PI3K activation during incipient pancreatic neoplasia induces the disassembly of these anchoring complexes, and therefore represents a druggable target to inhibit pancreatic cell migration and invasion through restoration of hemidesmosomes.

Pancreatic ductal adenocarcinoma (PDAC) has a dismal survival rate. Patients are diagnosed at either a local invasive or metastatic stage, but will ultimately dye within five years after diagnosis. This dismal prognosis is first the result of a late diagnosis. Symptoms appear when the primary tumor has already spread out and specific diagnosis tools such as imaging techniques or biomarkers enabling the detection of early lesions are not available yet. Dissemination to distant organs occurs early, as revealed by using mouse models that spontaneously develop PDAC and reproducibly recapitulate both preneoplastic and neoplastic changes observed during human PDAC [1]. Such genetically engineered models express in the pancreas a mutated (activated) form of the Kras oncogene, the priming mutation occurring in 90% of human PDAC cases. The use of elegant gene reporter approaches that specifically allow the follow-up of mutated Kras cells, has recently enabled to demonstrate that already preneoplastic cells have the potential of migrating into the stroma and have the appearance of mesenchymal cells that have undergone through epithelial-to-mesenchymal transition [1]. When detached from the primary tumor, such preneoplastic cells are observed even prior to the detection of lesions at the primary site, and are capable of reaching the blood circulation. Although circulating preneoplastic cells are at this stage probably not competent to form metastases, their detection in the plasma could be a good indicator of the population that should merit a closer survey. Importantly, preneoplastic cell detachment from the basement membrane and migration and invasion through the stroma represent early steps during pancreatic carcinogenesis (Fig. 1). This implies that specific structures anchoring epithelial cells to the basement membrane disrupt. In complex epithelia (e.g. skin), such anchorage structures are provided by hemidesmosomes (hereafter called HDs). However, in simple epithelia (e.g. exocrine pancreas) the existence of HDs and hence their role in the process of carcinogenesis remained unexplored.

Laval and al. [2] report the presence in the simple pancreatic epithelium of mature type-1 HDs located at the basal pole of duct cells, in contact with the anchoring basal membrane. These pancreatic type-1 HDs are composed of the integrin α6β4, plectin, and the bullous pemphigoid antigens BP180 and BP230. Importantly, type-1 HDs are shown to disassemble very early during pancreatic tumorigenesis (Fig. 1), while integrin β4 and BP180 are delocalized to the lateral membrane. In this new environment, integrin β4 is known to interact with growth factor receptors, to get phosphorylated and then to facilitate cell migration and invasion instead of being an anchorage factor. Interestingly, delocalization of integrin β4 together with its interacting partner plectin, had previously been observed in human preneoplastic lesions, where these proteins are overexpressed and could represent (plectin) interesting imaging tool target to diagnose PDAC earlier [3,4]. What are the mechanistic insights underlying type-1 HD breakdown during incipient pancreatic neoplasia? Transgenic mouse models have revealed that the phosphoinositide 3-kinase (PI3K) signal initiates the development of preneoplastic lesions [5]. Laval et al. [2] describe an interesting scenario highlighting the critical role of PI3K in regulating type-1 HD turnover, and reconciling the ideas that both PI3K activation and migration/invasion of preneoplastic cells occur as soon as the earliest phases of pancreatic carcinogenesis [2, 5]. The first step of type-1 HD breakdown actually requires a PI3K-dependent induction of MMP-9 expression and subsequent MMP-9-mediated cleavage of the extracellular domain of BP180. The cleavage of BP180 results in the destabilization of its interaction with both integrin α6β4 and BP230. New complexes comprising only integrin α6β4 and plectin form labile type-2 HDs which are probably maintained until integrin β4 gets phosphorylated by PI3K, which has been elsewhere reported to induce integrin β4 dissociation from its partner plectin [6]. Conversely, inhibiting PI3K activity in pancreatic cancer cells with specific pharmacological inhibitors decreases cell migration and invasion through the restoration of mature type-1 HDs. A similar inhibition of PI3K activity and consequent restoration of type-1 HD can be achieved through activation of the G protein-coupled somatostatin receptor sst2 in epithelial cancer cells of pancreatic as well as of skin or oropharynx origin. The somatostatin-sst2 signaling pathway thus appears as a natural system ensuring the integrity of epithelia through the maintenance of type-1 HDs. This is of particular interest for PDAC because sst2 expression has been shown to be extinguished in human preneoplastic and PDAC lesions [7, 8]. The restoration of the somatostatin-sst2 system in pancreatic tumors therefore offers a new therapeutic opportunity to limit pancreatic cancer cell migration and invasion. In this perspective, the results of a clinical gene-therapy trial (NCT01274455, TherGap Trial, Investigator L. Buscail, promoter CHU Toulouse) aimed at expressing in pancreatic cancer the receptor sst2 and an engineered kinase (dck::umk) enabling the *in-cellulo* activation of the chemotherapeutic drug gemcitabine are eagerly awaited.
